# *Polygala tenuifolia*: a source for anti-Alzheimer’s disease drugs

**DOI:** 10.1080/13880209.2020.1758732

**Published:** 2020-05-19

**Authors:** Xinxin Deng, Shipeng Zhao, Xinqi Liu, Lu Han, Ruizhou Wang, Huifeng Hao, Yanna Jiao, Shuyan Han, Changcai Bai

**Affiliations:** a Ningxia Medical University Pharmacy College, Key Laboratory of Hui Ethnic Medicine Modernization, Ministry of Education, Ningxia Research Center of Modern Hui Medicine Engineering and Technology, Yinchuan, P. R. China; b College of Basic Medical Sciences, Jilin University, Changchun, Jilin, P. R. China; c Key Laboratory of Carcinogenesis and Translational Research (Ministry of Education), Department of Integration of Chinese and Western Medicine, Peking University Cancer Hospital and Institute, Beijing, P. R. China

**Keywords:** Polygalasaponin XXXII, tenuifolin, polygalacic acid, senegenin, tenuigenin, neuroprotective effects, multitarge

## Abstract

**Context:**

Alzheimer’s disease (AD) is a chronic neurodegenerative disease that originates from central nervous system lesions or recessions. Current estimates suggest that this disease affects over 35 million people worldwide. However, lacking effective drugs is the biggest handicap in treating AD. In traditional Chinese medicine (TCM), *Polygala tenuifolia* Willd. (Polygalaceae) is generally used to treat insomnia, memory dysfunction and neurasthenia.

**Objective:**

This review article explores the role of *P. tenuifolia* and its active components in anti-Alzheimer’s disease.

**Methods:**

Literature for the last ten years was obtained through a search on PubMed, SciFinder, CNKI, Google Scholar, Web of Science, Science Direct and China Knowledge Resource Integrated with the following keywords: Polygala tenuifolia, polygalasaponin XXXII (PGS 32), tenuifolin, polygalacic acid, senegenin, tenuigenin, Alzheimer’s disease.

**Results:**

*Polygala tenuifolia* and its active components have multiplex neuroprotective potential associated with AD, such as anti-A*β* aggregation, anti-Tau protein, anti-inflammation, antioxidant, anti-neuronal apoptosis, enhancing central cholinergic system and promote neuronal proliferation.

**Conclusions:**

*Polygala tenuifolia* and its active components exhibit multiple neuroprotective effects. Hence, *P. tenuifolia* is a potential drug against Alzheimer’s disease, especially in terms of prevention.

## Introduction

Alzheimer’s disease (AD), also called senile dementia, is a chronic neurodegenerative disease that originates from central nervous system lesions or recessions. The number of people with AD globally is predicted to exceed one billion in 2050 (Anand et al. 2014; Khoury et al. 2017). According to the ‘World Alzheimer Report 2015’, the number of patients with AD in China is the highest in the world, and it is equivalent to approximately 1 out of every 20 elderly people (Prince et al. 2015). AD has affected not only the elderly population but also the middle-aged group (Castellani et al. 2010). An increase in the number of patients is accompanied by a remarkable social pressure. In the United States alone, more than $170 billion is used to treat AD per year (Reitz and Mayeux 2014). Although AD is a major health problem worldwide, the pathogenesis of AD has only begun to be explored gradually in the future.

**Figure 1.**
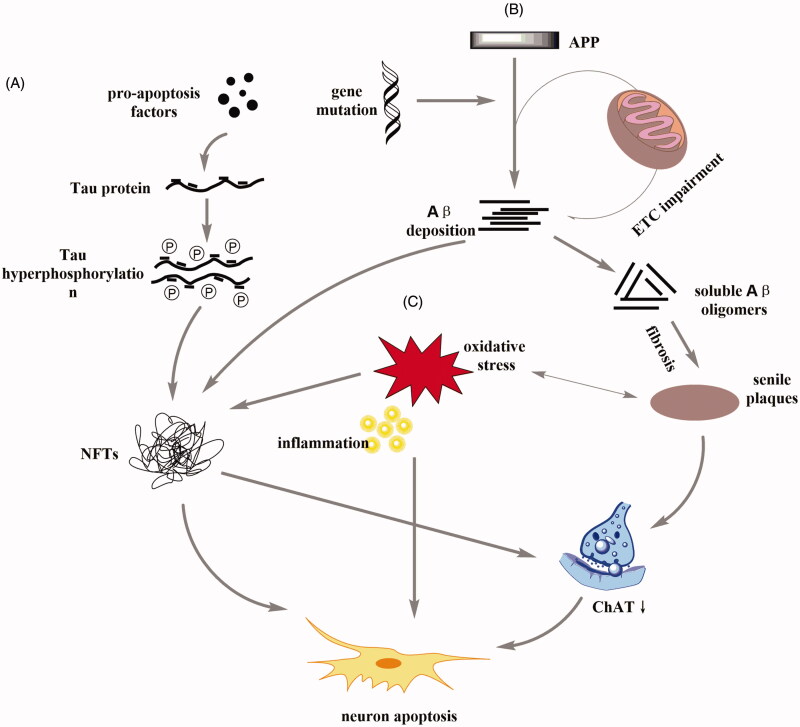
Schematic diagram of common pathogenesis of AD. (A) The presence of pro-apoptotic factors can promote the phosphorylation of tau protein to form neurofibrillary tangles (NFTs). NFTs can directly promote neuronal apoptosis, or indirectly lead to neuronal apoptosis by affecting the activity of ChAT. (B) The electron transport chain (ETC) is a key step in the energy release of the mitochondria. ETC dysfunction and gene mutations trigger metabolic disorders of amyloid precursor protein (APP) and promote Aβ deposition. Aβ deposition can both promote NFTs and affect the content of ChAT through the formation of senile plaques. (C) Inflammation and oxidative stress can directly stimulate neurons and participate in the development of NFTs and senile plaques.

Pathological hallmarks of AD include senile plaque, neurofibrillary tangles (NFTs), synaptic loss and neuronal dysfunction (Hong et al. 2016; Das and Yan 2017; Ishizuka and Hanamura 2017). Abnormal aggregation of Aβ protein in senile plaques was previously considered to triggers the pathological cascade of AD, suggesting that the accumulation of Aβ in insoluble Aβ fibrils promotes the development of AD (Gouras et al. 2015). The NFTs in AD composed of paired helical filaments aggregated by hyperphosphorylated tau (p-tau) (Liu et al. 2010). Tau, a microtubule associated protein, is a major component of the paired helical filaments observed in the brains of AD patients, which normally binds to tubulin to promote microtubule stability (Amemori et al. 2015). p-Tau is positively correlated with AD development in clinical experiments (Zhang et al. 2017). Furthermore, oxidative stress interferes with AD development by inducing plaque and NFTs formation, subsequent synaptic and neuronal loss (Chen and Zhong 2014; Friedemann et al. 2015). The role of central cholinergic damage in AD development has also been widely investigated. The loss of cholinergic neurons, the synthesis and release of choline acetyltransferase (ChAT), and decreased sensitivity to acetylcholine receptors cause a cognitive dysfunction in the brains of patients with AD (Pu and Wang 2010; Ferreira-Vieira et al. 2016). The above pathogenesis of AD is shown in Figure 1.

In response to this disease, drugs such as galantamine, tacrine, memantine, donepezil and rivastigmine have been used in clinical settings (Piemontese 2017). Although these medicines can alleviate AD-associated symptoms, some adverse events, such as liver toxicity, diarrhoea and vomiting also appear (Grutzendler and Morris 2001; Lane et al. 2018). Unfortunately, several promising drugs have failed in phase II/III clinical trials. Therefore, the selection of multi-target drugs will be a potentially effective strategy for AD treatment. Some active extracts or components from TCM have properties of neurological disorders treatment because of its multicomponent and multitarget properties and low toxicity (Wu et al. 2011; Wang et al. 2013).

**Figure 2.**
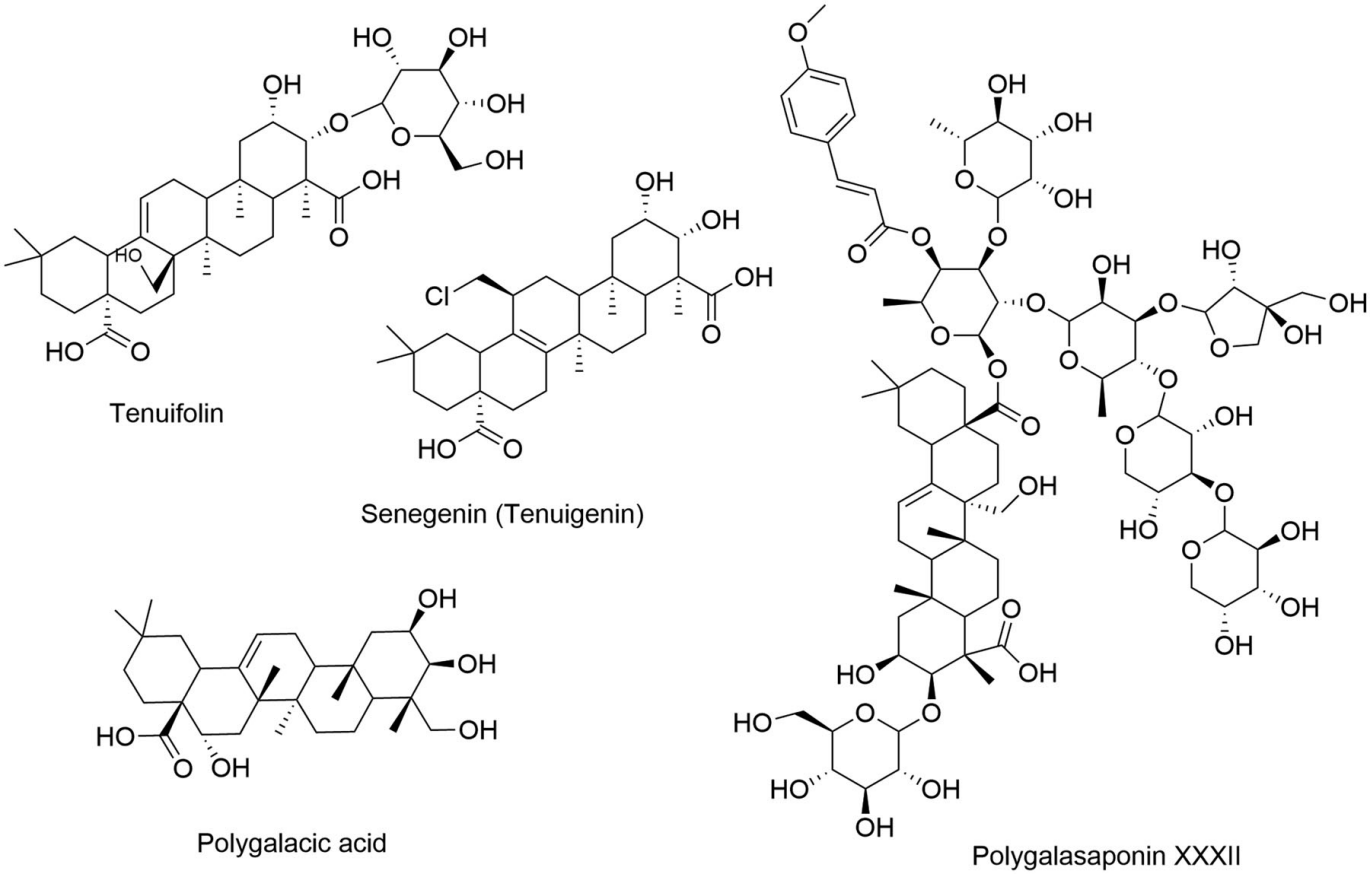
Chemical structures of active compounds from *Polygala tenuifolia*.

*Polygala tenuifolia* Willd. (Polygalaceae), also known as Yuan Zhi, has been reported to be enriched with triterpene saponins, onjisaponins and polygalasaponins (Jin et al. 2014), and it also exhibits protective effects on the central nervous system and is frequently used to treat memory dysfunction, insomnia and neurasthenia (Zhang et al. 2016). As the main pharmacologically active components of *P. tenuifolia*, polygalasaponins, including polygalasaponin XXXII (PGS 32), tenuifolin, polygalacic acid and senegenin (tenuigenin), have been shown to possess multiplex neuroprotective potential associated with AD, such as anti-β-amyloid (Aβ) aggregation (Jia et al. 2004; Park et al. 2019), anti-Tau (Xu et al. 2012), anti-inflammation (Cheong et al. 2011; Wang et al. 2017), antioxidant (Zhang et al. 2011), enhancing central cholinergic system, anti-neuronal apoptosis (Li et al. 2015), promote neuronal proliferation (Park et al. 2008; Zhu et al. 2016). The chemical structures are presented in Figure 2.

## *Neuroprotective effects of* Polygala tenuifolia

Accumulated evidence suggests that *P. tenuifolia* extract and many active components [such as PGS 32, tenuifolin, senegenin (tenuigenin) and polygalacic acid] exert neuroprotective effects (Table 1), including anti-A*β* aggregation, anti-Tau, anti-inflammation, antioxidant, enhancing central cholinergic system, anti-neuronal apoptosis and promote neuronal proliferation.

**Table 1.** The effect of active agents in *Polygala tenuifolia* against Alzheimer's disease.

Active agents	Molecular formula	Method	Model	Dose	Effect	Reference
Polygalasaponin XXXII	C_79_H_118_O_38_	*In vitro*	Primary cortical neurons, PC12 cells	1–100 *µ*g/ml	Heighten the survival rate of neurons	(Zhou et al. 2016)
		*In vivo*	Kunming mice (18–20 g, male), C57BL/6J mice (18–20 g), Wistar rats (230–260 g)	7.5–30 mg/kg	Significant improvement scopolamine-induced memory impairment by upregulated Tr*κ*B and p-Tr*κ*B level	(Zhou et al. 2016)
Senegenin	C_30_H_45_ClO_6_	*In vitro*	Primary cortical neurons from Neonatal SD rats	2 *µ*M	Activate PI3K/Akt signalling pathway to elevate neuron survival	(Pi et al. 2016)
		*In vivo*	SD rats (280 ± 30 g)	18.5–74 mg/kg	Reduces accumulation of A*β*_1–40_ and abnormal tau phosphorylation in the brain by modulating the PI3K/Akt pathway	(Chen et al. 2015)
Polygalacic acid	C_30_H_48_O_6_	*In vitro*	–	–	–	–
		*In vivo*	Kunming mice (18–22 g, male)	3–12 mg/kg	Improves the cholinergic system anti-oxidative stress and anti-neuritis, and enhances cognitive function by reducing AChE activity while increasing ChAT activity	(Guo et al. 2016)
Tenuifolin	C_36_H_56_O_12_	*In vitro*	PC12 cells	1–40 *µ*g/ml	Reduces Aβ_25–35_-induced cytotoxicity in PC12 cells	(Liu et al. 2015)
		*In vivo*	C57BL/6J mice (26–30 g, male)	3–9 mg/kg	Improve the memory dysfunction caused by Aβ_25–35_	(Liu et al. 2015)

‘–’ means no relevant literature found

### Anti-Aβ

Senile plaque, abnormal aggregation of Aβ, is an important pathological feature of AD, and it is the foremost origin during the development of neurocytotoxicity. The formation and clearance of Aβ in the brain of normal people are in equilibrium, and this balance is broken in the AD patient’s brain due to excessive Aβ. Aβ has two forms of existence, dissolution and deposition. Aβ in the dissolved state promotes the growth of neurites and the survival of neurons. On the contrary, it causes axonal degeneration and neuronal degeneration (Pike et al. 1992).

*Polygala tenuifolia* extract and several components derived from *P. tenuifolia* have exhibited anti-Aβ effects. For example, tenuifolin which was separated from the crude extract of *P. tenuifolia* inhibits Aβ secretion without changing the ratio of Aβ_1–40_ and Aβ_1–42_ in African green monkey COS-7 cells which transfected with either APP695 cDNA and the Swedish mutation, the effect may be related to inhibition of the β-site APP cleaving enzyme (Lv et al. 2009). Meanwhile, tenuigenin can reduce the level of Aβ in human neuroblastoma SH-SY5Y APP695 cells via inhibiting the activity of BACE1 (Jia et al. 2004). In the AD rat model, tenuigenin inhibits Aβ_1–40_ aggregation, decrease phosphorylation level of Tau^Ser396^ by down-regulating of ubiquitin expression and up-regulating activity of ubiquitin ligase E3 and 26S proteasome at doses of 37.0 and 74.0 mg/kg. This result indicates that the role of tenuigenin in combating AD may be related to the ubiquitin–proteasome pathway (Chen et al. 2015).

In addition to reducing the aggregation of Aβ, *P. tenuifolia* also has a therapeutic effect on Aβ-induced neurotoxicity. Senegenin protect PC12 cells from Aβ_25–35_-induced cytotoxicity by increasing neurite number, average length and maximum length as well as the expression of MAP2 and Gap-43 (Jesky and Chen 2016). PSM-04, a crude extract from the root of *P. tenuifolia*, reduces Aβ aggregation and neurotoxicity caused by oligomeric Aβ, inhibits hydrogen peroxide (H_2_O_2_)-induced oxidative stress and Aβ-induced neuronal apoptosis, increases the expression of superoxide dismutase-2 protein in brain tissues of 5xFAD (Tg) mice (Park et al. 2019).

### Anti-Tau

Another hallmark of AD is NFTs, and highly phosphorylated tau proteins are the major content of NFTs. Tau protein kinases, such as glycogen synthase kinase-3, cyclin-dependent protein kinase-5 and mitogen-activated protein kinases (MAPK), play a key role in the development of phosphorylation of tau protein (Martin et al. 2013). Studies have shown that *P. tenuifolia* can regulate the imbalance between tau protein kinases and phosphatases. In AD rat model induced by Aβ_1–42_, tenuigenin significantly decreased the expression of GSK-3β and CDK-5 while up-regulated the activity of PP-1 and PP-2A and inhibited the abnormal phosphorylation of tau in hippocampus (Chen et al. 2012a). Another study indicates that the tenuigenin protects neurons against Aβ_1–40_-induced tau phosphorylation by down-regulating PKA protein expression, increasing PP-2A activity and inhibiting hyperphosphorylation of the MAPT Ser^396^ in rat brain neurons (Xu et al. 2012). *O*-Linked *N*-acetylglucosamine (*O*-GlcNAc) glycosylation is a dynamic post-translational modification of nucleocytoplasmic proteins which regulate by glycosyltransferase *O*-GlcNAc transferase (OGT) and glycoside hydrolase *O*-GlcNAcase (OGA). In the presence of OGT, O-GlcNAc is transferred from the UDP-Glc NAc donor via the *O*-glycosidic bond and ligated to the hydroxyl group of serine or threonine. In contrast, OGA can eliminate the protein modification of *O*-GlcNAc (Zhu et al. 2014). Previous studies have shown that *O*-GlcNAc can inhibit the phosphorylation of tau (Lefebvre et al. 2003). In P12 cells, tenuigenin can increase the level of *O*-GlcNAc glycosylation in a dose-dependent manner, restrain phosphorylation levels of tau proteins at Ser^396/404^, Ser^202^, Thr^205^, Thr^212^ and Thr^217^ (Chen et al. 2012b).

### Anti-inflammation

Activated microglia play a crucial role in neuroinflammation. Aβ can induces microglia to release inflammatory cytokines by binding to receptors on the plasma membrane of microglia (Yu and Ye 2015). The water extract of *P. tenuifolia* roots (2, 4 and 8 μg/mL) inhibited the protein expression of nitric oxide (NO), nitric oxide synthase (iNOS), prostaglandin E2 (PGE2), interleukin-1β (IL-1β), tumour necrosis factor-α (TNF-α) and cyclooxygenase-2 (COX-2) in lipopolysaccharide (LPS)-treated BV2 microglia. And the water extract of *P. tenuifolia* roots also blocked the translocation and transcriptional activity of NF-κB by impeding IκB-α degradation and inhibiting TLR4 and MyD88 expression (Cheong et al. 2011). Tenuigenin inhibits LPS-induced PGE2 and NO production and decrease iNOS and COX-2 gene expression in RAW 264.7 macrophages by inhibiting MAPK/NF-kB and activating the Nrf2/HO-1 signalling pathways (Lv et al. 2016). Tenuigenin also inhibits inflammatory cytokines (IL-1β, IL-6 and TNF-α) and PGE2 expression via activation of Nrf2-mediated HO-1 signalling pathway in LPS-activated murine BV2 microglia cells (Wang et al. 2017).

### Antioxidant

Oxidative stress is one of the most important factors in the development of AD. When the body is subjected to harmful stimuli, excessive production of high active molecules (Reactive Oxygen Species, ROS) leads to imbalance between oxidation and antioxidant defences which results in oxidative stress (Yan et al. 2013). Excessive ROS can cause protein damage, induce oxidative stress and ultimately lead to cell death (Perry et al. 1998; Stringfellow et al. 2014). Evidence has shown that H_2_O_2_ can also accelerate apoptosis (Pierce et al. 1991). As a catalytic enzyme, superoxide dismutases (SOD) can reduce O_2_− to H_2_O_2_ (Arteel 2003). Glutathione peroxidase (GSH-Px) is another antioxidant enzyme that protects cells by catalysing the reduction of peroxides (Beutler 1972). Tenuigenin has been found to protect rat hippocampal neurons against streptozotocin-induced oxidative stress, neuronal damage and cognitive dysfunction by increasing the SOD and GSH-Px activities, down-regulating 4-hydroxy-2-nonenal adducts levels and inhibiting the phosphorylation of tau proteins (Huang et al. 2018). PSM-04, an extract from the root of *P. tenuifolia*, has neuroprotective effects via inhibiting ROS generation induced by H_2_O_2_, increasing the expression of SOD-2 and brain derived neurotrophic factor (BDNF), reducing the neurotoxicity induced by oligomeric A*β*_1–42_ and suppressing amyloid plaques in the hippocampus (Park et al. 2019). Tenuigenin (10 μM) protects SH-SY5Y cells against 6-hydroxydopamine-induced damage and apoptosis by increasing SOD and GSH, MMP levels, inhibiting caspase-3 activation, and stimulating TH expression (Liang et al. 2011). Tenuigenin also inhibited the formation of reactive oxygen species promoted by methylglyoxal, in rat hippocampal neurons in primary culture (Chen et al. 2010). These evidences suggest that *P. tenuifolia* may be effective in treating AD associated with oxidative stress.

### Enhancing central cholinergic system

Acetylcholinesterase (AChE) is an essential enzyme in nerve conduction that terminates the signal transmission by catalyses the hydrolysis of the neurotransmitter acetylcholine (ACh). AChE also participates in cell development and maturation, promotes neuronal development and regeneration (Soreq and Seidman 2001). AChE has become a considerable biomarker for diagnosing AD, because of reduction in AChE activity in brains of AD patients (Meena et al. 2019). And reducing acetylcholinesterase while increasing the availability of ACh in central cholinergic synapses could be beneficial to alleviating the symptoms of AD (Wu et al. 2018). Thus, AChE inhibitors are most promising for treatment of AD (Davidsson et al. 2001). BT-11 (extract of dried root of *P. tenuifolia*) restrained AChE activity in a dose-dependent and non-competitive manner (IC_50_ value: 263.7 μg/mL) in Aβ_1–42_-induced SD rats (Park et al. 2002). The results of the Y-maze task show that tenuigenin can improve the learning and memory ability of mice in the model group, and it can also reduce the AChE activity and lower malondialdehyde level, while increasing SOD activities (Huang et al. 2013). Polygallic acid is a hydrolysate of triterpenoid saponin which has the function of regulating cholinergic activity. It can significantly increase ACh and ChAT expression, decrease AChE actility in the hippocampus and frontal cortex (Guo et al. 2016).

### Anti-neuronal apoptosis

Neuronal apoptosis plays a vital role in central nervous system development and many neurodegenerative diseases. Moreover, caspase-mediated pathway was closely related to the progression of apoptosis. When this pathway is activated, caspase mobilizes the death programme via wrecking key agents of the cellular infrastructure and triggering factors that mediate cell injure. Cytochrome c (Cyt c), an electron mediator in mitochondria, also activates caspase-mediated pathways when it is transferred from damaged mitochondria to cytoplasm (Friedlander 2003). The Bcl-2 family is also involved in apoptosis. Bax and Bcl-2 are two major regulators of apoptosis. Bax can promote apoptosis, whereas Bcl-2 restrains apoptosis. The higher ratio of Bax/Bcl-2 implicates that apoptosis occurs (Yang and Korsmeyer 1996). Tenuigenin protects PC12 cells against Aβ_25–35_-induced apoptosis. After tenuigenin (50, 100, 200 μmol/L) for 24 h, the survival rate of PC12 cells was significantly increased, the apoptosis rate and Cyt c expression level were lower than those in the model group (*p* < 0.01). And the ratio of Bcl-2/Bax increased to 0.64, 1.29 and 1.84, respectively (Yang et al. 2013). In primary rat hippocampal neuronal cultures, tenuigenin protected neurons from methylglyoxal-induced excitotoxicities in a dose-dependent manner (1–4 μg/mL). Moreover, western blot assays indicate that tenuigenin can increase the expression of Bcl-2 and down-regulate the expression of Bax and caspase-3 (Chen et al. 2010).

### Promote neuronal proliferation

The therapeutic strategy to promote neuron regeneration and inhibiting neuron apoptosis may be promising in treatment of AD. BDNF is a considerable neurotrophic factor involved in neuron plasticity, development, survival and differentiation of the neurons via activating tropomyosin-related kinase (Trk) receptors (Binder and Scharfman 2004). At a dose of 5 μg/mL, senegenin promoted neuritogenesis with a notable increase in the number of neurites, mean length, and maximum length by up-regulating MAP2 and Gap-43 expression or inhibition of ASK1 and JNK signalling pathways (Jesky and Chen 2016). Another research suggests that senegenin exerts neuroprotective effects may be associated with the PI3K/Akt signalling pathway (Pi et al. 2016). In addition, *P. tenuifolia* extract can also promote the nerve growth factor release in astroglial cells (Yabe et al. 2003). PGS32 significantly increased BDNF content by up-regulation the phosphorylation of ERK, CREB and synapsin I (Xue et al. 2009). PGS32 can also ameliorate against scopolamine-induced memory impairments in AD mice model via up-regulating the level of the p-TrkB, enhanced high frequency stimulation-induced long-term potentiation in the dentate gyrus of rats, and protecting of neurons from damage caused by glutamate and ROS (Zhou et al. 2016).

## Conclusions

AD is a chronic neurodegenerative disease with a high incidence rate and a large number of patients, which seriously impact on the patients’ quality of life. However, no effective drugs have been found to prevent and treat this disease (Li et al. 2016). *Polygala tenuifolia* and its active components have exhibited a wide range of pharmacologic effects *in vitro* and *in vivo*, such as neuroprotective, immunomodulatory, anti-inflammatory, hepatoprotection, antioxidative, antibacterial and antitumor effects. However, despite many promising pre-clinical reports, there are no toxicological studies and clinical trials have been reported to date. Further studies should focus on the toxicological and pharmacokinetics aspects of the potential anti-AD agents, and that will help researchers determine the safe dose and range of medication, provide a basis for the structural transformation of new drugs, and ensure the safety of clinical medications. The quality control is another major challenge in *P. tenuifolia* application. Due to the differences in habitat, harvest time and processing methods, the treatment effect of *P. tenuifolia* is also uneven (Su et al. 2018). Therefore, it is considerably important to formulate drug quality control standards and clarify processing conditions and provide uniform and reliable medicinal materials for clinical use.
